# Extracellular matrix-based biomaterials as adipose-derived stem cell delivery vehicles in wound healing: a comparative study between a collagen scaffold and two xenografts

**DOI:** 10.1186/s13287-020-02021-x

**Published:** 2020-11-27

**Authors:** Héctor Capella-Monsonís, Andrea De Pieri, Rita Peixoto, Stefanie Korntner, Dimitrios I. Zeugolis

**Affiliations:** 1 Regenerative, Modular & Developmental Engineering Laboratory (REMODEL), Biomedical Sciences Building, National University of Ireland Galway (NUI Galway), Galway, Ireland; 2 Science Foundation Ireland (SFI) Centre for Research in Medical Devices (CÚRAM), Biomedical Sciences Building, National University of Ireland Galway (NUI Galway), Galway, Ireland; 3 Proxy Biomedical Ltd., Spiddal, Ireland; 4 Regenerative, Modular & Developmental Engineering Laboratory (REMODEL), Faculty of Biomedical Sciences, Università della Svizzera Italiana (USI), Lugano, Switzerland

**Keywords:** Human adipose-derived stem cell delivery, Collagen scaffolds, Decellularised grafts, Wound healing

## Abstract

**Background:**

Stem cell therapies represent a promising tool in regenerative medicine. Considering the drawbacks of direct stem cell injections (e.g. poor cell localisation), extracellular matrix-based biomaterials (e.g. scaffolds and tissue grafts), due to their compositional biofunctionality and cytocompatibility, are under investigation as potential stem cell carriers.

**Methods:**

The present study assessed the potential of three commercially available extracellular matrix-based biomaterials [a collagen/glycosaminoglycan scaffold (Integra™ Matrix Wound Dressing), a decellularised porcine peritoneum (XenoMEM™) and a porcine urinary bladder (MatriStem™)] as human adipose-derived stem cell delivery vehicles.

**Results:**

Both tissue grafts induced significantly (*p* < 0.01) higher human adipose-derived stem cell proliferation in vitro over the collagen scaffold, especially when the cells were seeded on the basement membrane side. Human adipose-derived stem cell phenotype and trilineage differentiation potential was preserved in all biomaterials. In a splinted wound healing nude mouse model, in comparison to sham, biomaterials alone and cells alone groups, all biomaterials seeded with human adipose-derived stem cells showed a moderate improvement of wound closure, a significantly (*p* < 0.05) lower wound gap and scar index and a significantly (*p* < 0.05) higher proportion of mature collagen deposition and angiogenesis (the highest, *p <* 0.01, was observed for the cell loaded at the basement membrane XenoMEM™ group). All cell-loaded biomaterial groups retained more cells at the implantation side than the direct injection group, even though they were loaded with half of the cells than the cell injection group.

**Conclusions:**

This study further advocates the use of extracellular matrix-based biomaterials (in particular porcine peritoneum) as human adipose-derived stem cell delivery vehicles.

**Graphical abstract:**

Comparative analysis of a collagen scaffold (Integra™ Matrix Wound Dressing) and two tissue grafts [decellularised porcine peritoneum (XenoMEM™) and porcine urinary bladder (MatriStem™)] as human adipose-derived stem cells carriers 
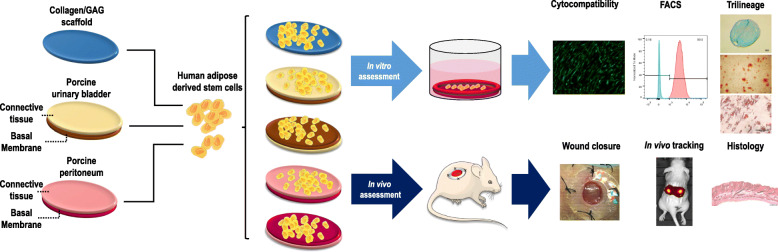

## Background

Stem cell-based therapies emerged as the pinnacle of regenerative medicine and as the most promising therapeutic solution to a broad spectrum of injuries and degenerative conditions. With global revenue of over US$1 billion per annum, thousands of products on the market and many more at advanced phases of clinical trials and industrial pipeline [1], the therapeutic application of stem cells in regenerative medicine in undeniable. Despite the high prospects and considerable advances of stem cell-based therapies, many limitations still need to be addressed for their efficient use in clinic, including poor cell engraftment at the implantation side and the large number of cells required for therapeutic effect [2].

Biomaterials, by providing stem cell anchoring sites, can be employed as stem cell carriers to maximise their retention at the side of implantation. The ideal stem cell biomaterial carrier must be cytocompatible, provide mechanical support, ensure cell function after transplantation, promote autologous cell infiltration and be resorbable. In the quest of the ideal stem cell biomaterial carrier, extracellular matrix (ECM)-based biomaterials (e.g. extracted collagen scaffolds and decellularised tissue grafts) are favoured due to their inherent cytocompatibility, low immunogenicity and tunable mechanical properties. In particular, decellularised tissue grafts hold great promise, largely attributed to the multifunctional composition of their preserved ECM that no man-made device will ever match. Unfortunately, the ideal tissue graft for soft tissue repair and regeneration remains elusive, considering their scattered clinical outcomes (e.g. Permacol™ in hernia repair [3, 4], CorMatrix® in paediatric cardiovascular surgery [5, 6] and Strattice® in breast reconstruction [7, 8] have shown both positive and negative results).

Appropriately decellularised and processed porcine peritoneum contains a broad range of ECM molecules (e.g. collagen type I, collagen type III, collagen type IV, fibronectin, elastin, laminin) and growth factors [e.g. vascular endothelial growth factor (VEGF), fibroblast growth factor 2 (FGF-2)] [9–12], which are well-known stem cell function regulators (e.g. promote stem cell adhesion, proliferation, migration and differentiation) [13, 14] and wound healing promoters (e.g. promote cell proliferation and angiogenesis in vivo) [15, 16]. Further, it has a well-established high cytocompatibility and low immunogenicity in vitro [17, 18] and high cell proliferation and low immune response in vivo [19]. Despite all these positive attributes, porcine peritoneum has neither been assessed in wound healing context nor as a stem cell carrier.

Herein, we ventured to assess the potential of decellularised porcine peritoneum (XenoMEM™; alone and) as a human adipose-derived stem cell carrier in a splinted nude mouse wound healing model, investigating also the effect that its components (connective tissue and basement membrane layers) may have on this application. As controls (in addition to sham and cells alone), we used a commercially available, also bilayer, decellularised porcine urinary bladder graft (MatriStem™) and a collagen/glycosaminoglycan (GAG) scaffold (Integra™ Matrix Wound Dressing; both alone and with cells), as they have an established clinical history even in challenging wound healing incidents (e.g. burn treatment: MatriStem™ [20], Integra™ Matrix Wound Dressing [21]).

## Materials and methods

### Materials

The decellularised porcine peritoneum (XenoMEM™) was provided by Viscus Biologics LLC (USA) in freeze-dried state. The decellularised porcine urinary bladder (MatriStem™) was purchased from ACell® (USA) in a freeze-dried state. The collagen/GAG scaffold (Integra™ Matrix Wound Dressing) was purchased from Integra Life Sciences Corporation (USA) in wet state in phosphate-buffered saline (PBS). Human adipose-derived stem cells were purchased from Lonza (UK). All chemical and consumables were purchased from Sigma-Aldrich (Ireland), unless otherwise stated.

### In vitro cytocompatibility assessment

Human adipose-derived stem cells (hADSCs, PT-5006, Lonza, UK) were expanded in alpha-minimal essential medium (α-MEM) GlutaMAX™ medium (Gibco, Ireland) supplemented with 10% foetal bovine serum (FBS), 1% penicillin/streptomycin (PS) and 5 ng/ml FGF-2 (PromoCell, Ireland) at 5% CO_2_ and 37 °C. Cells were used to passage 5 for all in vitro experiments and were seeded on the materials following standard protocols. Briefly, materials were cut in 1 cm^2^ pieces, placed at the bottom of 24-well plates and fixed with silicone O-rings to prevent their floating. Materials were then sterilised with 70% ethanol for 30 min and washed with PBS 3 times. ADSC were seeded on the Integra™ scaffolds and on both connective tissue (CT) and basement membrane (BM) sides of MatriStem™ and XenoMEM™ at a density of 25,000 cells/cm^2^ in α-MEM with 10% FBS and 1% PS and incubated at 5% CO_2_ and 37 °C for 3, 7 and 14 days, replacing the media every 3 days and using tissue culture plastic (TCP) as control. At each time point, cell viability and metabolic activity were assessed employing LIVE/DEAD® (ThermoFisher, Ireland) and alamarBlue® (ThermoFisher, Ireland) assays, respectively, as per manufacturer’s protocols. Proliferation and morphology were assessed by fixation of the cells with 4% paraformaldehyde (PFA) for 20 min at room temperature and subsequent staining of the cytoskeleton with 1:500 rhodamine-phalloidin (Life Technologies, Ireland) for 1 h and of the nuclei with 1:2000 Hoechst (Invitrogen, Ireland) for 5 min. Images of the stained cells were taken using an inverted fluorescence microscope (IX81, Olympus, UK) and nuclei were counted with ImageJ (NIH, USA) to quantify the proliferation of cells on the materials and TCP.

### Flow cytometry analysis

hADSCs were seeded at 25,000 cells/cm^2^ density on TCP, on the Integra™ scaffold and on both sides of the MatriStem™ and the XenoMEM™ as described above and cultured for 14 and 21 days in α-MEM with 10% FBS and 1% PS at 5% CO_2_ and 37 °C, replacing the media every 3 days. At each time point, cells were detached with 0.25% trypsin/ethylenediaminetetraacetic acid (EDTA) solution, filtered through a 40-μm cell strainer (ThermoFisher, Ireland), centrifuged at 300×*g* for 5 min, resuspended at 10^6^ cells/ml with 2% FBS and 0.05% sodium azide in PBS and kept on ice. One hundred microliters of each cell suspension (~ 10^5^ cells) were incubated with fluorescence labelled antibodies for the mesenchymal stem cells markers CD90^+^, CD73^+^, CD44^+^ (respective product codes 51-9007657, 51-9007649, 51-9007656, BD Bioscience, Ireland) and CD45^−^ (product code 46-0459-41, ThermoFisher, Ireland) and their correspondent isotype controls (CD90^+^, CD73^+^, CD44^+^ isotype cocktails, product codes 51-9007664, 51-9007655 , BD Bioscience, Ireland; CD45^−^ isotype, product code 46-4714-80, ThermoFisher, Ireland) for 30 min at 4 °C in dark. The cell suspensions were then centrifuged at 300×*g* for 5 min, washed in 2 ml of 2% FBS and 0.05% sodium azide solution and centrifuged as above. The supernatants were discarded by decantation and the cell pellets were resuspended in the remaining FBS and sodium azide solution with a vortex, and 5 μl of Sytox™ Blue (ThermoFisher, Ireland) were added to stain dead cells. Cell suspensions were then analysed using a BD FACS Canto™ II flow cytometer (BD Biosciences, Ireland). Analysis was carried out until 10,000 counts were reached and data were processed with the software FlowJo™ v10 (FlowJo™ LLC, USA).

### Trilineage differentiation analysis

For trilineage differentiation, 25,000 hADSCs per square centimetre were seeded on TCP, on the Integra™ scaffold and on both sides of the MatriStem™ and the XenoMEM™ as described above and were incubated at 5% CO_2_ and 37 °C in α-MEM with 10% FBS and 1% PS for 3 days. Osteogenic, adipogenic and chondrogenic differentiations were conducted following established protocols.

For osteogenic differentiation, cells on TCP and on the different materials were treated with α-MEM supplemented with 10% FBS, 1% PS, 100 nM dexamethasone, 50 μM ascorbic acid 2-phospate and 10 mM β-glycerophosphate disodium salt hydrate. The osteogenic media were replaced every 3 days. At days 7, 14 and 21, cells on TCP and the different materials were washed with PBS, treated with 0.5 M HCl and disrupted by scratching with a pipette tip. The solution was then collected, incubated overnight at 4 °C under agitation, centrifuged at 500×*g* to discard cell debris and the calcium of the supernatant was quantified with a calcium colorimetric assay (MAK022, Sigma-Aldrich, Ireland). Treated cells on TCP were also stained with 2% alizarin red solution after fixation with methanol as positive control of differentiation and images were taken with an inverted microscope (CKX41, Olympus, UK).

For adipogenic differentiation, cells on TCP and on the different materials were treated for 3 days with adipogenic induction media (Dulbecco’s modified Eagle medium, DMEM, high glucose supplemented with 10% FBS, 1% PS, 1 μM dexamethasone, 1 μM rosiglitazone, 0.5 mM 3-isobutyl-1-methyl-xanthine, 10 μg/ml insulin) and for 3 subsequent days with adipogenic maintenance media (10% FBS, 1% PS, 10 μg/ml insulin) in repeating cycles for 7, 14 and 21 days. At each time point, cells were washed in PBS and fixed with 4% PFA. All conditions were stained with oil red O staining solution for 5 min, washed with 60% propanol and washed 3 times with distilled water. Images of the stained cells on TCP were taken with an inverted microscope (CKX41, Olympus, UK), as quality control of differentiation. Ninety-nine percent propanol was poured on the samples to extract the oil red O stain, and the solution was transferred to Eppendorf tubes and centrifuged at 500×*g* for 5 min to remove cell debris. Optical density (OD) at 520 nm was read to measure the quantity of released stain in the solution. Blanks with only materials were run to subtract any noise signal.

For chondrogenic differentiation, cells on TCP and on the different materials were treated with chondrogenic media [DMEM high glucose supplemented with 1% PS, 100 nM dexamethasone, 1x ITS+1 liquid media supplement (insulin, transferrin, sodium selenite, linoleic-bovine serum albumin), 40 μg/ml L-proline, 50 μg/ml ascorbic acid 2-phosphate and 10 ng/ml transforming growth factor β3 (TGF-β3, R&D Systems, UK)] for 7, 14 and 21 days, changing the media every 3 days. To form pellets, 5 × 10^5^ cells were suspended in chondrogenic media, centrifuged at 300×*g* for 5 min and incubated as the rest of conditions. At each time point, sulphated glycosaminoglycans (GAGs) where quantified using a colorimetric kit (Blyscan™, Biocolor, UK) as per manufacturer’s protocol. Pellets at 7, 14 and 21 days were fixed with 4% PFA for 1 h at 4 °C and incubated in 15% and 30% sucrose for 1 h in each solution at 4 °C under mild agitation. The pellets were then embedded in OCT™ compound (Tissue-Tek®, Sakura®, The Netherlands), snap-frozen and cryosectioned (CM1850 Cryostat, Leica BioSystems, UK). Cryosections (~ 7 μm in thickness) were stained with Alcian Blue and Nuclear Fast Red solution and imaged with inverted microscope (CKX41, Olympus, UK).

### In vivo stem cell delivery in a splinted wound model

Animal studies were carried out under approval of the Animal Care Research Ethics Committee of the NUI Galway (Approval number 15/DEC/07). A well-established in the literature splinted nude mouse wound healing model for cell transplantation was used [22–24]. In brief, 50–60-day-old athymic mice were anaesthetised with isoflurane, the skin of the dorsal area was disinfected with iodine and two full-thickness wounds of 5 mm in diameter were created using a punch biopsy. Silicone splints of 6 mm internal diameter and 12 mm external diameter were fixed around each wound with superglue and secured to the skin with 6–0 nylon suture stiches (Ethicon, Ireland) to prevent skin contraction. Animals (*n* = 6 for each group) were randomly assigned to one treatment in both wounds as follows: no treatment control (sham), topical application of 10^6^ hADSC in 50 μl of PBS, each one of the materials alone (in tissue grafts, both the CT and the BM were placed facing the exterior of the wound) and each of the materials loaded with hADSCs (in tissue grafts, both the CT and the BM were loaded with cells and placed facing the exterior of the wound). All materials were applied as discs of 5 mm in diameter in wet state. As the tissue grafts were provided in freeze-dried state, they were incubated in sterile PBS for 30 min at room temperature prior to application. hADSCs were seeded on the materials 24 h before implantation at a density of 2.6 × 10^6^ cells/cm^2^, and at the surgery, 5 mm in diameter pieces were carrying approximately 5 × 10^5^ cells. Cell-loaded materials were applied to the wounds with the cell-loaded side facing the exterior of the wound. In both cells alone and cell-loaded materials groups, cells were stained with fluorescent solution (Vybrant™ DiD, ThermoFisher, Ireland) for 20 min at 37 °C prior to implantation or seeding, respectively, for their fluorescent tracking in vivo [25]. After application of the treatment, wounds were protected with the securement dressing Tegaderm™ (3 M, USA) and a cast was applied for the full duration of the study.

### In vivo cell tracking

At days 3, 7, 10 and 14, animals treated with cells were anaesthetised with isoflurane and fluorescent tracking of the cells was carried out using an in vivo imaging system (IVIS® Lumina III, PerkinElmer, UK). The Living Image® software (IVIS® Lumina, PerkinElmer, UK) was used to calculate the intensity of the fluorescence of labelled cells at the wound areas.

### Wound closure rate analysis

Animals were anaesthetised with isoflurane and pictures of the wounds were taken at days 3, 7, 10 and 14 with an iPad Pro (Apple, USA). Images were analysed and wound area was accurately calculated using the WoundWiseIQ (Med-Compliance IQ Inc., USA) software. The wound closure rate was calculated using the following equation: % Wound closure = [(Day 0 wound area − Day *X* wound area)/Day 0 wound area] × 100.

### Histology analysis

At day 14, animals were euthanised by CO_2_ overdose and tissue samples were harvested with an 8 mm biopsy punch and fixed in 4% PFA for 24 h at 4 °C. Cross-sections of 5 μm in thickness were prepared from paraffin blocks after processing of the tissue in a tissue processor (Excelsior AS, ThermoFisher, Ireland). The sections were deparaffinised in xylene and hydrated in descending concentrations of ethanol. Slides were stained using standard protocols for haematoxylin-eosin, Masson’s Goldner trichrome and picrosirius red. The sections were then dehydrated in ascending solutions of ethanol and xylene and mounted on DPX mounting medium. Images were captured with an Olympus VS120 digital scanner using the OlyVIA software (both Olympus Corporation, UK). For picrosirius red, an Olympus BX51-microscope (Olympus, UK) was used equipped with a circular light polariser (Olympus, UK) to obtain polarised light images.

Masson’s trichrome and haematoxylin/eosin images were used to calculate the wound gap, scar index and epidermal thickness. Briefly, 4 non-consecutive sections with a separation of 2 sections between them were used per sample to analyse the wound gap with ImageJ (NIH, USA) software using the line tool and measuring its length. Scar index was calculated in 4 non-consecutive sections with a separation of 2 sections between them; the scar tissue was outlined using the polygonal outline tool in ImageJ (NIH, USA) and the area was then measured. Dermal thickness was estimated by drawing a line to the skin orientation and measuring its length; 4 dermal thickness values were obtained per image in 4 non-consecutive sections, with a separation of 2 sections between them. The scar index was calculated by dividing the wound area between the average dermal thickness. The thickness of neo-formed epidermis was evaluated using ImageJ line tool (NIH, USA); 3 high-power fields per sample and 5 measurements of the epidermal thickness per field were obtained.

Picrosirius red images in 4 non-consecutive sections, with a separation of 2 sections between them were used to measure total collagen and mature collagen deposition. Briefly, bright field images were used to calculate the section area with ImageJ (NIH, USA) and total collagen was calculated by measuring the area of polarised images after applying the correspondent threshold. Using the channel split tool of ImageJ (NIH, USA), the area of the red channel, related to deposited mature collagen, was normalised to the correspondent total deposited collagen; 3 sections were analysed per sample.

### Immunohistochemistry analysis

Immunohistochemistry of paraffin embedded sections was carried out to assess the formation of blood vessels and the presence of hADSCs in the wound area. Briefly, sections were processed through heat antigen retrieval with citrate buffer at pH 6.5 in a pressure cooker for 3 min after dewaxing in xylene and re-hydration in descending ethanol solutions. For angiogenesis assessment, sections were incubated in blocking buffer, consisting of PBS with 5% of normal goat serum and 0.01% of Triton X-100, for 1 h at room temperature. After 3 washes in PBS, samples were incubated with rabbit anti-CD31 antibody (ab28364, Abcam, UK) for 30 min at room temperature at a 1:50 dilution in blocking buffer. After further 3 washes with PBS, sections were incubated with an Alexa Fluor 488 goat anti-rabbit antibody (A11008, ThermoFisher, Ireland) in blocking buffer at 1:200 dilution for 1 h at room temperature, washed 3 more times in PBS and mounted with ProLong™ Gold antifade mountant with DAPI (ThermoFisher, Ireland). Images of the sections were taken with an inverted fluorescence microscope (IX81, Olympus, UK) and analysed with ImageJ (NIH, USA) by counting the cells and measuring the area of formed blood vessels, which were normalised to the section area.

### Statistical analysis

Data were analysed using the IBM SPSS Statistics (IBM Analytics, USA) software. One-way analysis of variance (ANOVA), followed by Fisher’s post hoc test, was employed after confirming normal distribution (Kolmogorov-Smirnov normality test) and equality of variances (Levine’s test for homogeneity of variance). For non-normal distributions or different variance, Mann-Whitney *U* test and Kruskal-Wallis test were employed. Significant difference was accepted at *p* < 0.05.

## Results

### Cytocompatibility analysis

**Fig. 1:**
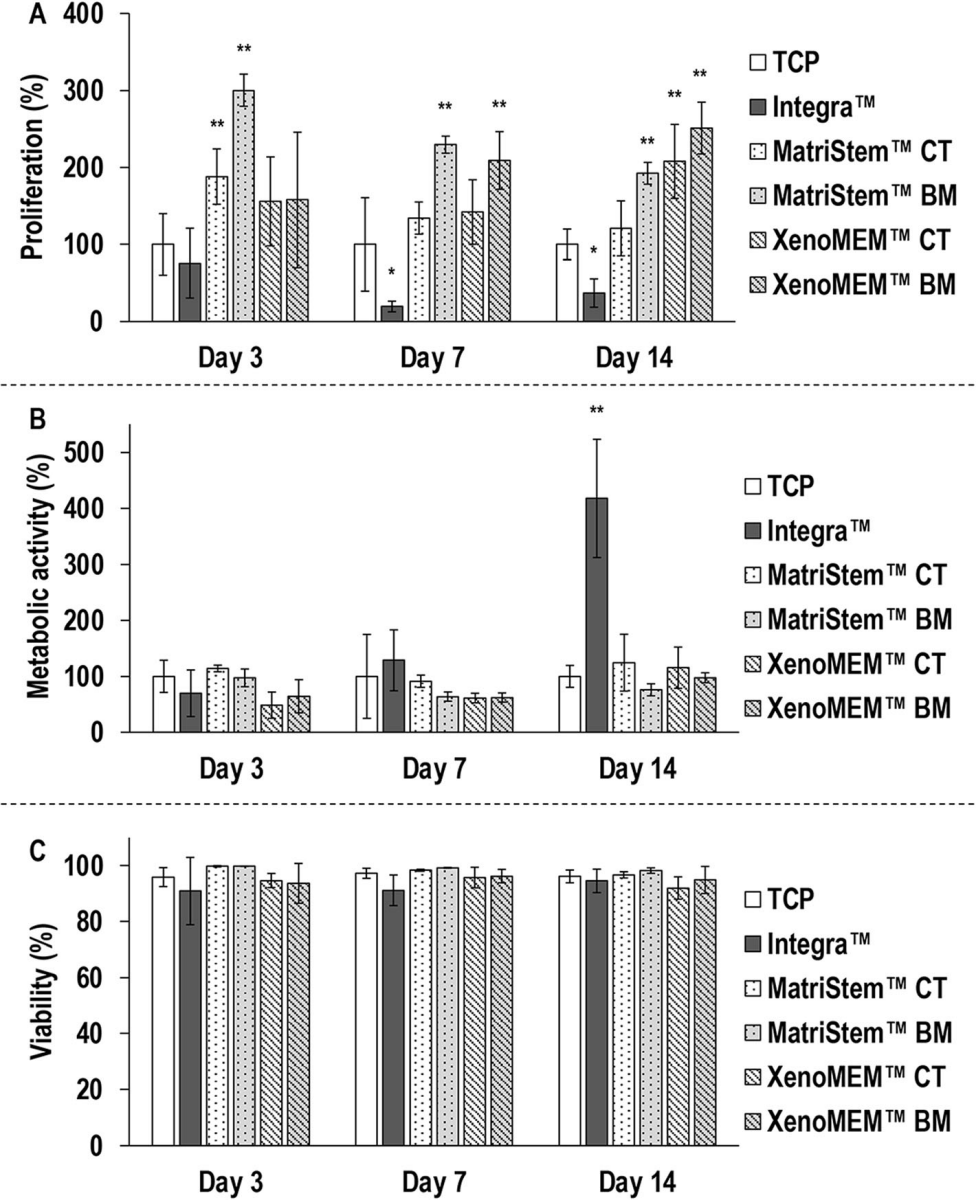
hADSC proliferation (**a**) was significantly higher on tissue grafts (in particularly on their basement membrane side) after 7 and 14 days than on TCP and on the collagen/GAG scaffold. hADSC metabolic activity (**b**) was significantly higher on the collagen/GAG scaffold after 14 days than on TCP and on the tissue grafts. hADSC cell viability (**c**) was not affected as a function of the different materials at any timepoint. Data presented as average ± standard deviation (*n* = 3). Asterisk indicates a significantly (*p* < 0.05) lower value than the TCP control and double asterisks indicate a significantly (*p* < 0.05) higher value than TCP

Qualitative cell morphology, proliferation (Supplementary Figure S1) and viability (Supplementary Figure S2) analyses revealed that hADSCs attached, spread and proliferated in higher rates on either side of both tissue grafts than on TCP and the Integra™ scaffold. Quantitative proliferation analysis (Fig. 1a) revealed that, in comparison to the control TCP, the Integra™ scaffold induced the lowest (*p <* 0.01) hADSCs proliferation at day 7 and day 14, whilst the highest (*p <* 0.05) hADSC proliferation was induced on both sides of MatriStem™ at day 3, on the BM sides of both tissue grafts at day 7 and on the BM side of MatriStem™ and both sides of XenoMEM™ at day 14. hADSCs metabolic activity analysis (Fig. 1b) revealed no significant (*p* > 0.05) differences between the groups at day 3 and day 7 and at day 14, the Integra™ scaffold induced the highest (*p* < 0.05) metabolic activity among all groups, whilst no significant (*p* > 0.05) differences were observed between the TCP and either side of both tissue grafts and between the tissue grafts. hADSC viability analysis (Fig. 1c) revealed no significant (*p* > 0.05) differences between the groups at any timepoint.

### Flow cytometry and trilineage differentiation analyses

Flow cytometry analysis (Supplementary Figure S3) revealed that most (> 99%) hADSCs on all groups expressed CD73, CD44 and CD90 and did not express CD45. Quantification of calcium deposition after osteogenic induction of the hADSCs (Supplementary Figure S4) revealed that, at all timepoints, the Integra™ scaffold and both tissue grafts exhibited significantly (*p* < 0.05) higher calcium deposition than the TCP. Oil red OD quantification after adipogenic induction of the hADSCs (Supplementary Figure S5) revealed that at day 7, the Integra™ scaffold exhibited the highest (*p* < 0.05) adipogenic potential and at days 14 and 21, the TCP and the Integra™ scaffold were significantly (*p* < 0.05 at day 14 and *p* < 0.01 at day 21) more adipogenic than both tissue grafts.

GAGs quantification after chondrogenic induction of the hADSCs (Supplementary Figure S6) revealed that at day 7, all conditions induced significantly (*p* < 0.05) higher chondrogenesis than TCP; at day 14, the CT side of XenoMEM™ induced the highest (*p* < 0.05) chondrogenesis; and at day 21, the pellet and the BM sides of MatriStem™ and XenoMEM™ induced the highest (*p* < 0.05) chondrogenesis.

### In vivo cell tracking analysis

**Fig. 2:**
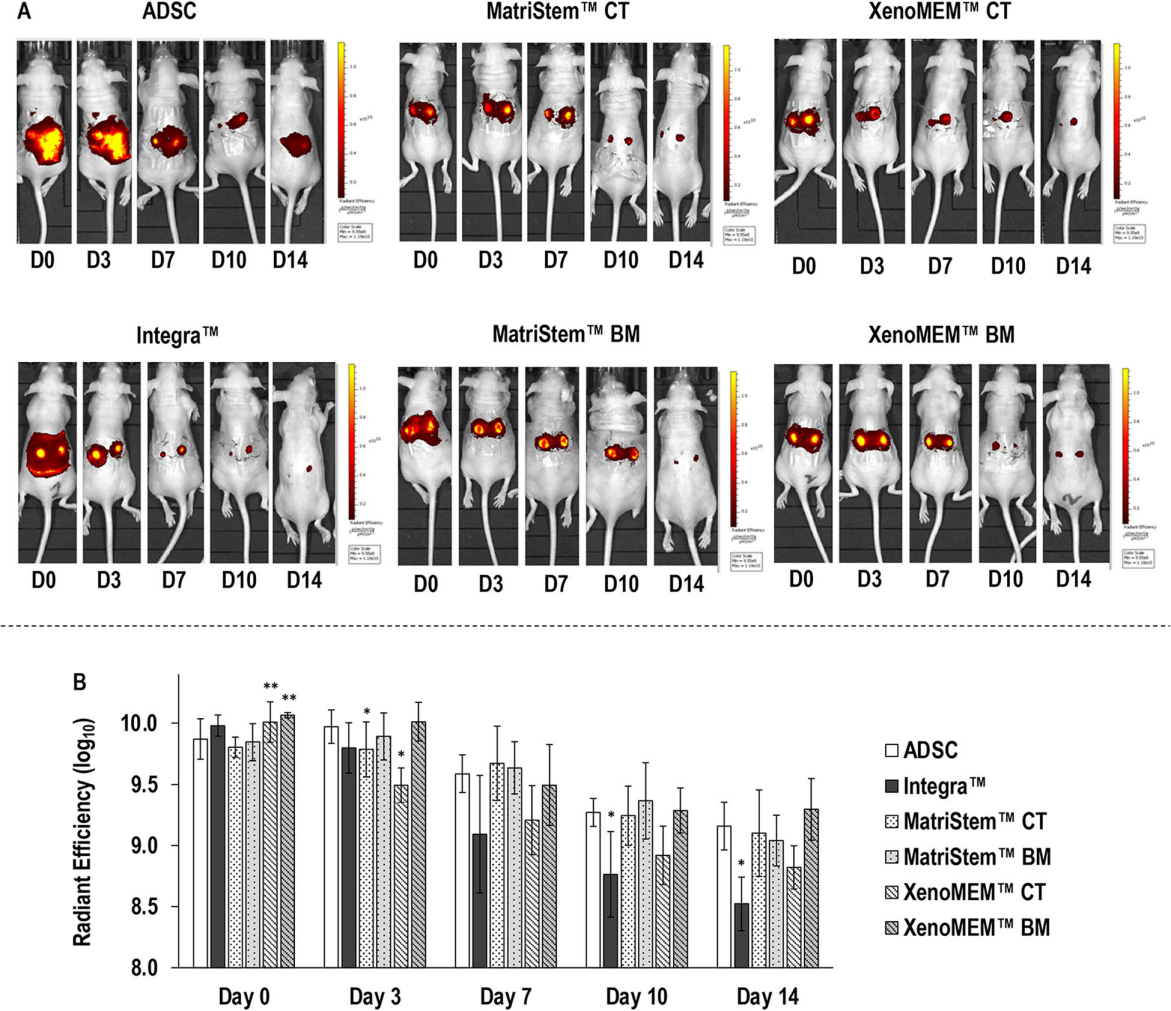
In vivo tracking of hADSC (**a**) revealed a disperse signal of the injected cells group and a localised signal for all materials groups, which was gradually lost in all groups. Quantification of radiant efficiency in the wound areas (**b**) made apparent a higher signal than the injected hADSC in both sides of XenoMEM™ at day 0, a lower signal in the CT sides of MatriStem™ and XenoMEM™ at day 3, and in Integra™ Matrix Wound Dressing at days 10 and 14. Data presented as average ± standard deviation (*n* = 6). Asterisk indicates a significantly (*p* < 0.05) lower value than the injected hADSC and double asterisks indicate a significantly (*p* < 0.05) higher value than the injected hADSC

Macroscopic analysis of fluorescent-labelled hADSCs (Fig. 2a) revealed that the cells of the cell injection group were dispersed around the dorsal area, whilst the cells that were delivered with the Integra™ scaffold and both tissue grafts were localised within the wounds. Further, for all groups, a gradual loss of signal was observed as a function of time (Fig. 2a). Quantification of radiance efficiency within the wounds (Fig 2b) revealed that at days 10 and 14 the Integra™ scaffold delivered hADSCs group exhibited significantly (*p* < 0.05) lower than the injected hADSCs group radiance efficiency within the wounds (at days 3 and 7, although radiance efficiency within the wounds was also lower, it was not significant). hADSCs that were delivered from the CT side of the XenoMEM™ (Fig. 2b) also showed significantly (*p* < 0.05) lower than the injected hADSCs group radiance efficiency within the wounds at day 3 (at days 7, 10 and 14, although radiance efficiency within the wounds was also lower, it was not significant). As a function of time, the radiance efficiency within the wounds was significantly (*p* < 0.05) reduced for all groups.

### Wound closure analysis

**Fig. 3:**
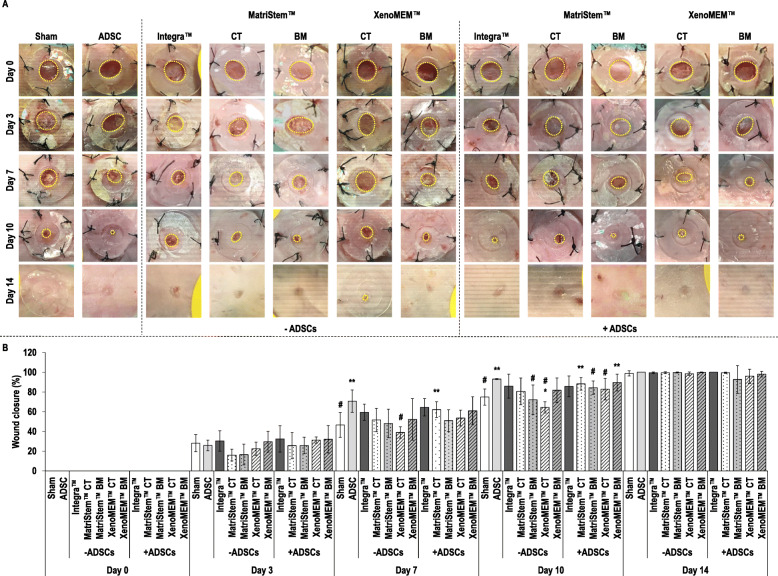
Macroscopic analysis (**a**) of the wounds (dashed yellow line) showed no complications nor scarring tissue during healing, all conditions reached wound closure after 14 days and hADSC accelerated the wound closure process. Quantification of wound closure (**b**) in the absence of hADSCs showed that the CT side of the XenoMEM™ induced significantly lower wound closure at day 7 in comparison to hADSC injection and at day 10 in comparison to sham and hADSC injection. The BM side of MatriStem™ BM also presented significantly lower wound closure than hADSC injection at day 10. Wound closure in the presence of hADSCs was significantly higher than the sham group for the hADSC injection and the CT side of MatriStem™ with hADSCs at day 7. The hADSC injection, the CT side of MatriStem™ with hADSCs and the BM side of XenoMEM™ with hADSCs showed significantly higher wound closure than sham at day 10. In comparison to hADSC injection, significantly lower wound closure was observed for the BM side of MatriStem™ and the CT side of XenoMEM™ at day 10. Data presented as average ± standard deviation (*n* = 6). Asterisk indicates a significantly (*p* < 0.05) lower value than the sham group, double asterisks indicate a significantly (*p* < 0.05) higher value than sham group and number sign indicates a significantly (*p* < 0.05) lower value than ADSC control

Macroscopic analysis of the wounds (Fig. 3a) revealed no apparent complication or excessive scarring in any of the conditions at any timepoint, all conditions resulted in an almost complete wound closure after 14 days and the use of hADSCs appeared to accelerate wound closure at a given timepoint.

Wound closure quantification (Fig. 3b) revealed no differences among groups at days 0, 3 and 14, whilst some differences were observed at days 7 and 10. Specifically, at day 7, the hADSC injection showed a significantly (*p* < 0.05) higher wound closure than the sham, the CT side of XenoMEM™ without hADSCs had significantly (*p* < 0.05) lower wound closure than the hADSC injection and the CT side of MatriStem™ with hADSCs had a significantly (*p* < 0.05) higher wound closure than the sham. At day 10, the hADSC injection showed a significantly (*p* < 0.05) higher wound closure than the sham, the BM of MastriStem™ without hADSCs presented a significantly (*p* < 0.05) lower wound closure than hADSC injection, the CT side of the XenoMEM™ without hADSCs had significantly (*p* < 0.05) lower wound closure than the sham and hADSC injection, the CT side of MatriStem™ and the BM side of XenoMEM™ with hADSCs had significantly (*p* < 0.05) higher wound closure than the sham and the BM side of MatriStem™ and CT side of XenoMEM™ with hADSCs had significantly (*p* < 0.05) lower wound closure than hADSCs injection (Fig. 3b).

### Histological analysis

**Fig. 4:**
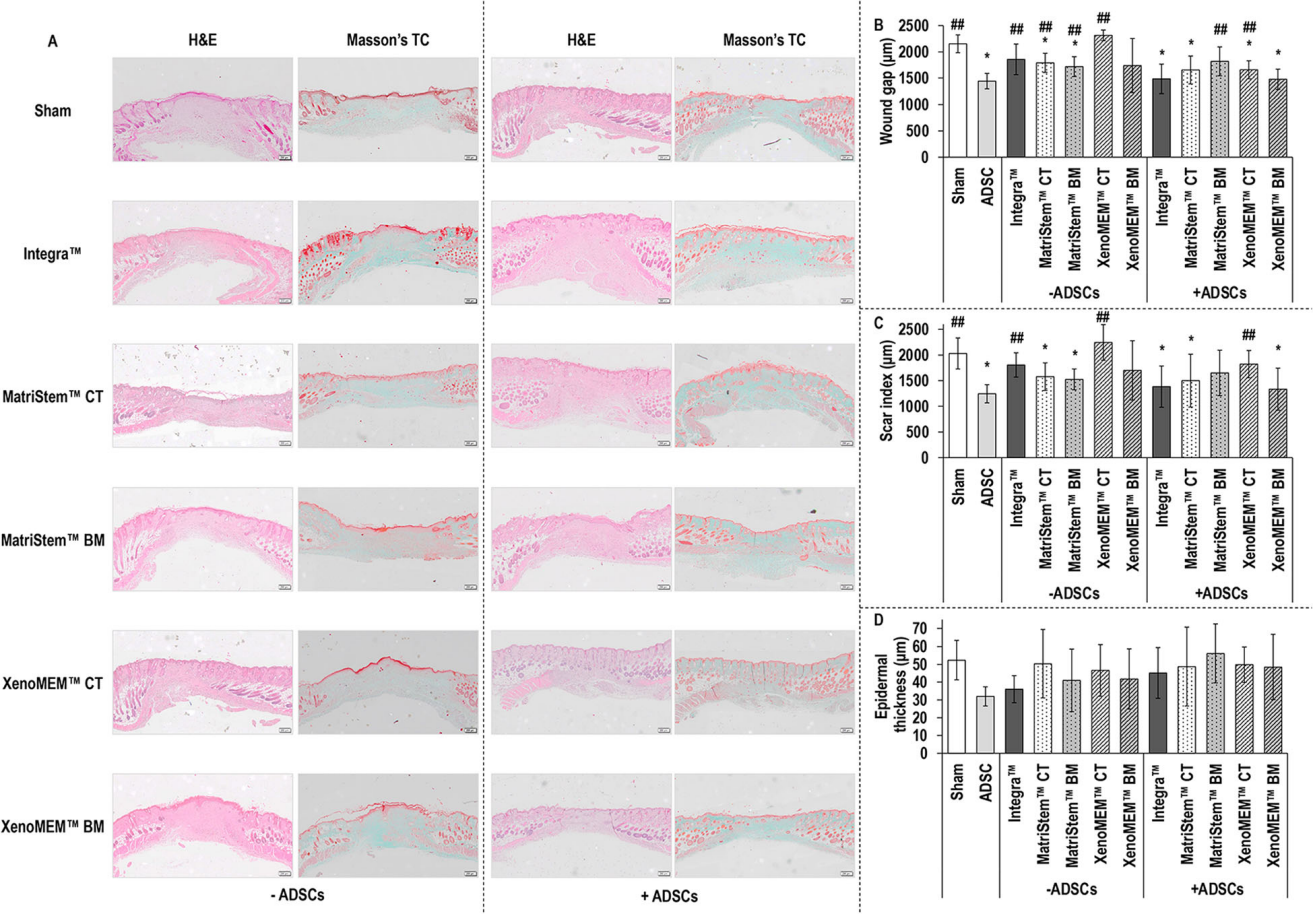
Haematoxylin/eosin (H&E) and Masson’s trichrome (Masson’s TC) stainings (**a**) revealed the gap in the dermis and *panniculus carnosus* filled with connective tissue and cells corresponding to the wound and made apparent that the use of hADSC decreased the wound gap. Scale bars 200 μm. Quantification of wound gap (**b**) showed that in the absence of hADSCs, a significantly lower wound gap was observed in both sides of MatriStem™ in comparison to the sham and significantly higher wound gap than the hADSC injection was found in all groups, but the BM side of the XenoMEM™. In the presence of hADSCs, all groups, but the BM side of the MatriStem™, significantly decreased the wound gap in comparison to sham and the BM side of the MatriStem™ and the CT side of the XenoMEM™ showed a significantly higher wound gap than the hADSC injection. Scar index quantification (**c**) showed that in the absence of hADSCs, a significantly lower scar index was observed in both sides of MatriStem™ in comparison to sham and significantly higher scar index than the hADSC injection was observed with the Integra™ scaffold and the CT side of the XenoMEM™. In the presence of hADSCs, the hADSC injection and hADSCs with the CT side of the MatriStem™ and the BM side of XenoMEM™ showed a significantly lower scar index than the sham and only the CT side of the XenoMEM™ showed a significantly higher scar index than hADSC injection. Epidermal thickness quantification (**d)** in the absence or presence of hADSC did not show any differences between groups. Data presented as average ± standard deviation (*n* = 6). Asterisk indicates a significantly (*p* < 0.05) lower value than the sham group and double number sign indicates a significantly (*p* < 0.05) higher value than ADSC group

Visual assessment of haematoxylin/eosin and Masson’s trichrome-stained sections of wounds at day 14 (Fig. 4a) revealed that the epidermis had been fully regenerated in all conditions, most of the materials had been remodelled [although some remnants were still present (Supplementary Figure S7)] and the wound gap had been reduced when hADSCs had been used. Quantification of the wound gap (Fig. 4b) revealed that in comparison to the sham group, in the absence of hADSCs, the CT and BM sides of the MatriStem™ showed significantly (*p* < 0.05) lower wound gap; in the presence of hADSCs, only the BM side of the MatriStem™ had similar (*p* > 0.05) wound gap to the sham group and all other groups had significantly (*p* < 0.05) lower wound gap. In comparison to the hADSC injection group, in the absence of hADSCs, only the BM side of the XenoMEM™ had a similar (*p* > 0.05) wound gap and all other groups presented a significantly (*p <* 0.05) higher wound gap, and in the presence of hADSCs, the BM side of the MatriStem™ and the CT side of the XenoMEM™ presented a significantly (*p* < 0.05) higher wound gap. When comparing each material without and with hADSCs, both sides of XenoMEM™ with hADSCs resulted in significantly (*p* < 0.05) lower wound gap than its without hADSCs counterpart. Quantification of the scar index (Fig. 4c) revealed that in comparison to sham, in the absence of hADSCs, the CT and BM sides of the MatriStem™ showed significantly (*p* < 0.05) lower scar index. In the presence of hADSCs, the BM side of the MatriStem™ and the CT side of the XenoMEM™ had similar (*p* > 0.05) scar index to the sham and all other groups had significantly (*p* < 0.05) lower scar index than the sham. In comparison to the hADSC injection, in the absence of hADSCs, the Integra™ scaffold and the CT side of XenoMEM™ showed a significantly (*p* < 0.05) higher scar index and, in the presence of hADSCs, only the CT side of XenoMEM™ had a significantly (*p* < 0.05) higher scar index. All materials with hADSCs, but the CT and BM sides of the MatriStem™, resulted in significantly (*p* < 0.05) lower scar index than their counterparts without hADSCs. Epidermal thickness analysis (Fig. 4d) revealed no significant (*p* > 0.05) differences between the groups and within the groups in the absence and presence of hADSCs.

**Fig. 5:**
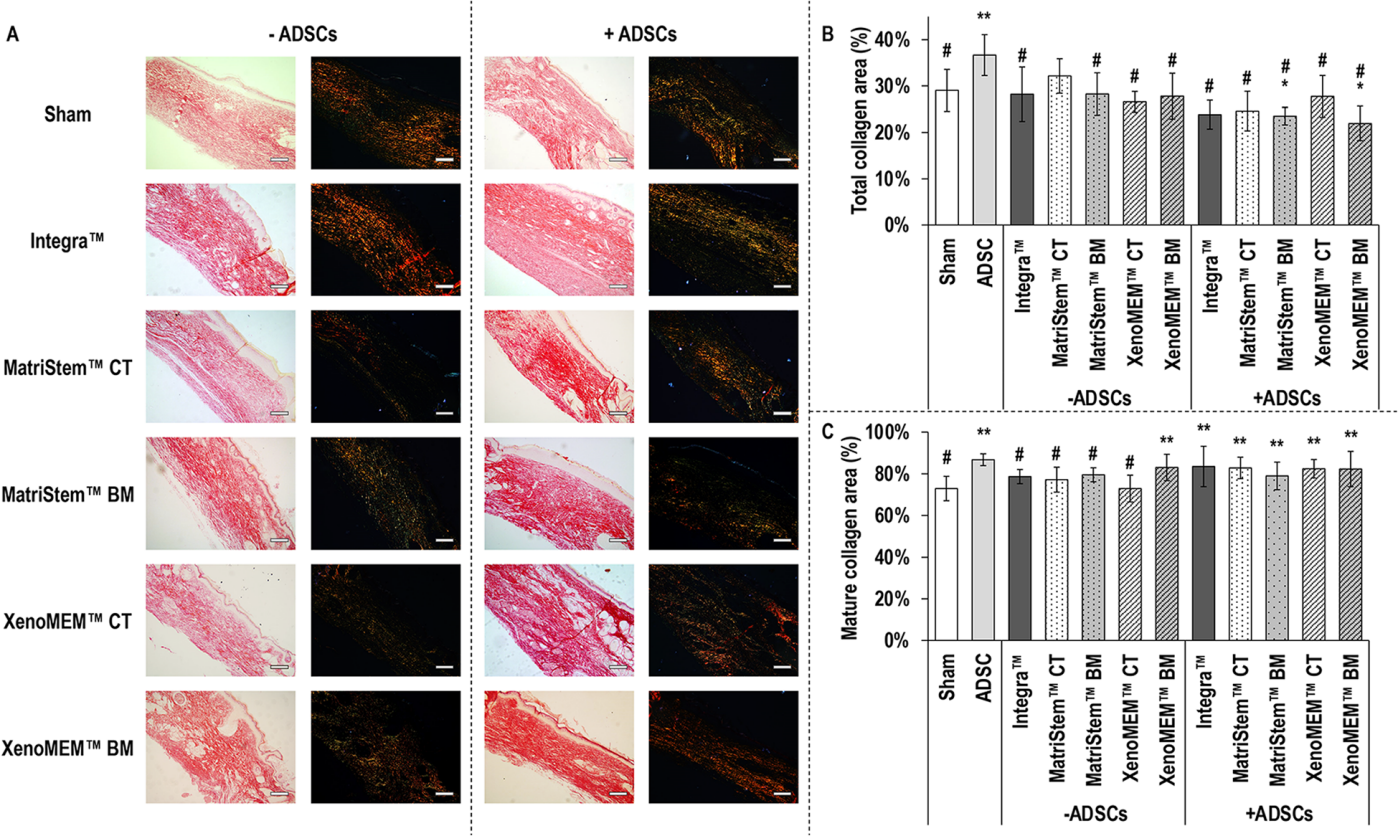
Polarised light microscopy of picrosirius red stained sections (**a**) showed the presence of disorganised mature (polarised red/yellow staining) and immature (polarised green staining) collagen in the wounds of all groups. Scale bars 50 μm. Quantification of total collagen (**b**) showed no differences among groups in the absence of hADSC and in the presence of hADSC, a significantly higher total collagen was observed in injected hADSC in comparison to the sham group and the significantly lower total collagen when cells were delivered with BM sides of the MatriStem™ and XenoMEM™ groups; in comparison to the hADSC injection, all groups, but the CT side of the MatriStem™ without hADSCs, showed a significantly lower total collagen area. Mature collagen quantification (**c**) in the absence of hADSC revealed a significantly higher amount of mature collagen in the BM side of XenoMEM™ in comparison to sham and all groups, but the BM side of the XenoMEM™ exhibited significantly lower mature collagen area than the hADSC injection. In the presence of hADSC, the injected cells and cells delivered with Integra™ Matrix Wound Dressing, the CT side of MatriStem™ and both sides of XenoMEM™ groups had significantly higher proportion of mature collagen than the sham group. Data presented as average ± standard deviation (*n* = 6). Asterisk indicates a significantly (*p* < 0.05) lower value than the sham group, double asterisks indicate a significantly (*p* < 0.05) higher value than sham group and number sign indicates a significantly (*p* < 0.05) lower value than ADSC group

Polarised light microscopy of picrosirius red stained sections (Fig. 5a) revealed the presence of disorganised collagen fibres in the wound area in all conditions, from which only the sham and the Integra™ scaffold in the absence of hADSCs and both sides of the XenoMEM™ in the presence of hADSCs induced matured collagen fibres. Total collagen area quantification (Fig. 5b) revealed that, in comparison to sham, in the absence of hADSCs, no significant (*p* > 0.05) differences were observed between the groups and, in the presence of hADSCs, the hADSC injection showed the highest (*p* < 0.05) total collagen area and the BM sides of the MatriStem™ and the XenoMEM™ showed significantly (*p* < 0.05) lower total collagen area. In comparison to the hADSC injection, all groups except the CT side of MatriStem™ without hADSCs had a significantly (*p <* 0.05) lower total collagen area. Materials with hADSCs had similar (*p >* 0.05) total collagen area to the materials without hADSCs, except for MatriStem™, which exhibited significantly (*p <* 0.05) lower total collagen area when loaded with hADSCs. Mature collagen quantification (Fig. 5c) revealed that in the absence of hADSCs, only the BM side of the XenoMEM™ had significantly (*p* < 0.05) higher mature collagen area in comparison to the sham group and all groups without hADSCs, but the BM side of the XenoMEM™, had a significantly (*p* < 0.05) lower mature collagen area compared to the hADSC injection. In the presence of hADSCs, all groups had significantly (*p* < 0.01) higher mature collagen area in comparison to the sham and presented no differences (*p* > 0.05) with the hADSC injection. Materials with hADSCs had similar (*p* > 0.05) mature collagen area to the materials without hADSCs, except of the CT side of the XenoMEM™ with hADSCs, which had significantly (*p* < 0.05) higher mature collagen area than its without hADSCs counterpart.

### Immunohistochemical analysis

**Fig. 6:**
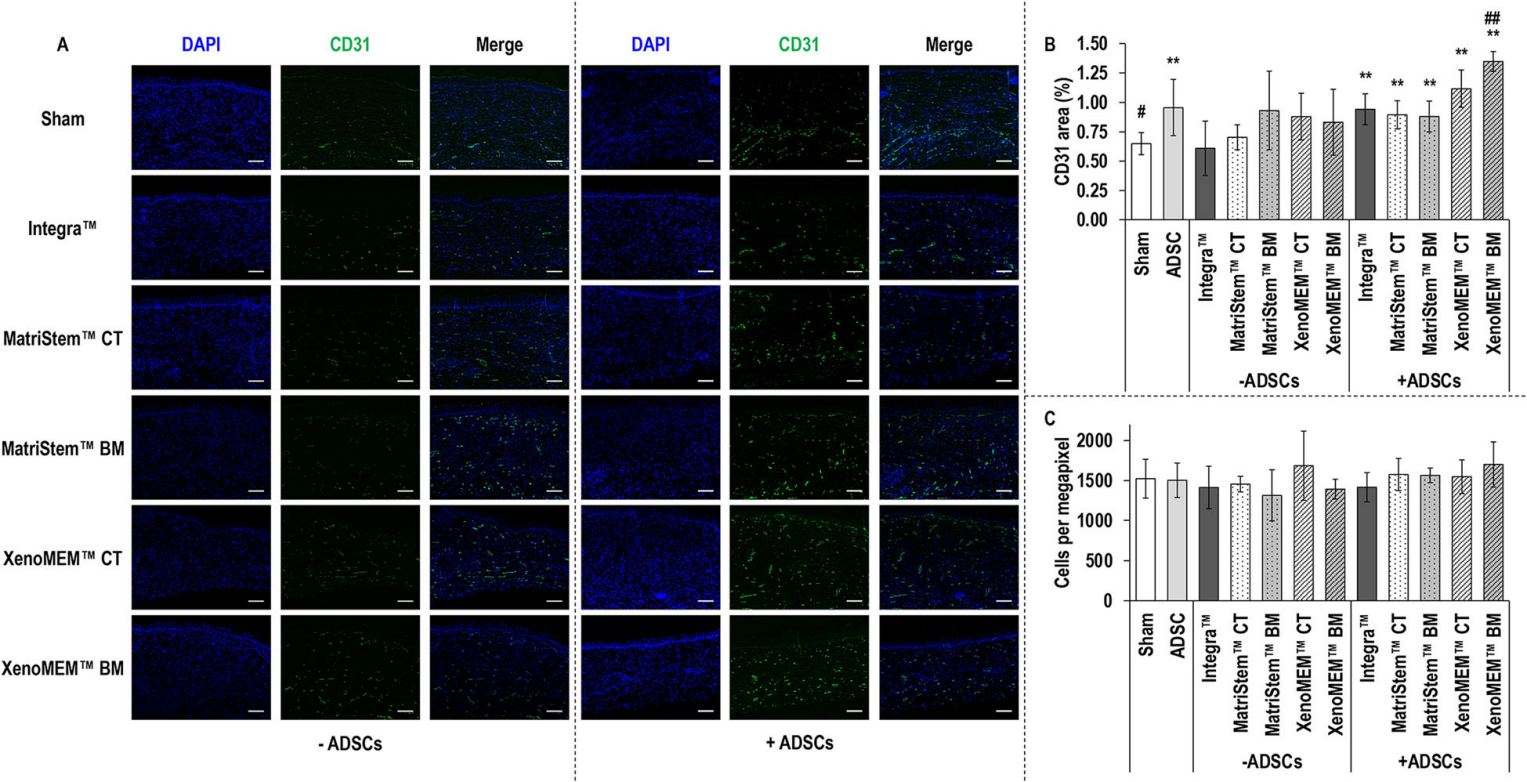
Immunohistochemistry analysis of sections (**a**) for CD31 (green) showed the formation of microvessels in all groups. DAPI (blue) staining (**a**) showed a homogenous dispersion of cells in the wound area in all conditions. Scale bars 100 μm. CD31 area quantification (**b**) showed no differences between groups in the absence of hADSC and in the presence of hADSC, a significantly higher area of CD31 in comparison to sham was observed in all groups and only the BM side of the XenoMEM™ showed significantly higher CD31 expression than the hADSC injection. Cell counting from DAPI stained sections (**c**) sections did not reveal any differences in cell density in the wounds between the groups in the absence or presence of hADSC. Data presented as average ± standard deviation (*n* = 6). Double asterisks indicate a significantly (*p* < 0.05) higher value than sham group, number sign indicates a significantly (*p <* 0.05) lower value than ADSC group and double number sign indicates a significantly (*p* < 0.01) higher value than ADSC group

Immunohistochemistry analysis of CD31 (Fig. 6a) revealed the formation of microvessels within the wound area in all conditions. Complementary image intensity analysis of CD31 (Fig. 6b) revealed in the absence of hADSCs no apparent differences (*p* > 0.05) between the groups, in the presence of hADSCs all groups exhibited significantly (*p* < 0.05) higher CD31 expression than the sham group and both sides of XenoMEM™ and Integra™ with hADSCs resulted in significantly (*p* < 0.01) higher CD31 expression than their without hADSCs counterparts. In comparison to the hADSC injection, only the BM side of XenoMEM™ with hADSCs showed a significantly (*p* < 0.01) higher CD31 expression. DAPI staining (Fig. 6a) revealed a homogenous distribution of cells in the wound area. Subsequent cell quantification in the wound area (Fig. 6c) revealed no differences (*p* > 0.05) between the groups in the absence and presence of hADSCs, and all materials with hADSCs, but the BM side of the XenoMEM™ (*p* < 0.05), resulted in similar (*p* > 0.05) cell number in the wound area than their without hADSCs counterparts.

## Discussion

Direct (stem) cell injections have failed to deliver consistent results in clinical practice, as the mode of administration neither protects nor localises the injected cell suspension at the side of implantation [26]. ECM-based biomaterials (e.g. extracted collagen scaffolds and decellularised tissue grafts) have the potential to act as excellent cell delivery vehicles, considering their well demonstrated cytocompatibility in vitro and remodelling capacity in vivo [27–29]. Unfortunately, the ideal ECM-based biomaterial remains elusive, largely attributed to their scattered clinical outcomes (‘from unacceptable to excellent’ [30]). Although in vitro and in vivo data advocate the potential of porcine peritoneum for regenerative medicine applications [17–19] and a few products are already clinically available (e.g. Meso BioMatrix®, DSM, for breast reconstruction; XenoMEM™, Viscus Biologics, hernia repair), its potential in wound healing and, in particular, as stem cell carrier has yet to be elucidated. Herein, we assessed the potential of porcine peritoneum (XenoMEM™, Viscus Biologics) as a human adipose-derived stem cell carrier in a splinted nude mouse wound healing model, taking also into consideration its layer-dependent composition (connective tissue and basement membrane layers). To ensure that the derived data will inform future clinical studies, we also used as control groups a collagen-based scaffold (Integra™ Matrix Wound Dressing, Integra Life Sciences Corporation) and another bilayer tissue graft (porcine urinary bladder, MatriStem™, Acell®), both with a well-documented clinical history in wound healing [20, 21].

Starting with in vitro cytocompatibility assessment on the various materials, it was found that hADSC proliferation was enhanced when seeded on the tissue grafts, which is in agreement with previous work with porcine urinary bladder [31] and is expected, due to the presence of growth factors (e.g. FGF-b, TGF-β1) in these matrices [32, 33] that are known to promote ADSC proliferation without affecting their stemness [34]. Furthermore, this proliferation was enhanced on the BM side of the tissue grafts, as has been previously observed with other cell types [11, 12], since BM is rich in collagen type IV and laminins [35] that are known to elicit such effect [36, 37]. When though the hADSCs were seeded on the collagen/GAG scaffold, a decreased proliferation and an increased metabolic activity were observed, indicative of cell stress, which can be probably attributed to the crosslinking method employed and is in agreement with previous publications using human bone marrow stem cells [38].

In general, none of the materials assessed affected the stemness and multilineage potential of the hADSC, as has been shown before for porcine urinary bladder [39] and collagen/GAG [40] devices. With respect to osteogenic potential, all materials induced osteogenesis after 21 days in culture, as has been shown before for urinary bladder [41] and collagen/GAG [40] devices using different stem cell populations. Although during adipogenic differentiation the lipid production was reduced at day 21 in comparison to day 14 in all tissue grafts, such reduction has been previously attributed to hADSC donors’ characteristics/conditions [42] or to the detachment of mature differentiated hADSC after long culture periods [43]. With respect to chondrogenic differentiation, it is worth noting that the BM sides of the tissue grafts exhibited significantly higher chondrogenic capacity even over the pellet culture that is considered the gold standard in in vitro setting [44]. It is worth noting that protocols similar to this study induced chondrogenesis in hADSC only in combination with TGF-β3 and/or other growth factors (e.g. FGF-18, IGF-1 BMP-6) [45–48]. This BM side preferential chondrogenic differentiation of the hADSCs may again be attributed to the composition of this tissue layer [e.g. laminin-1, collagen type IV and fibronectin have been shown to improve chondrogenesis in human bone marrow stem cells [37]].

Moving on to the preclinical assessment, it was evidenced that all materials retained more cells at the side of implantation than the cell injection approach, despite the fact that only half of the cells that were used in the direct injection approach were loaded on the materials. In fact, XenoMEM™ presented a higher signal at day 0, which is indicative of a higher presence of cells and therefore loading efficiency. Further, among the materials assessed, the collagen/GAG scaffold lost fluorescent signals the fastest, which we attribute to the potential cytotoxicity of the material that was observed in vitro or to the absence of as many cell attachment sites as the tissue grafts offer. Similar results have been reported before in the literature with a range of tissue grafts, cell populations and preclinical models (e.g. hADSC delivered by a porcine small intestinal submucosa graft to a rat ventral model [49], hADSC delivered by a decellularised porcine nucleus pulposus device to a rabbit intervertebral disc degeneration model [50], rat bone marrow stem cells delivered by a porcine decellularised meniscus materials to a full-thickness rat meniscus defect model [51]). The gradual loss in fluorescent signal observed in all conditions could be also attributed to the loss of scar tissue after 2 weeks, as has been suggested before in the same model [52, 53].

Histology and immunohistochemistry analyses showed the absence of a fibrotic response, as indicated by a lower scar index than the sham and no increase of cellularity in the wounds. In general, cell-loaded materials, even though they were loaded with half of the cells that were delivered through the direct injection approach, exhibited low levels of total collagen, high levels of mature collagen and high angiogenesis potential, which further advocate the paracrine antifibrotic effect, regenerative/remodelling capacity and vascularisation competence of ADSCs [54–56]. The injected hADSCs resulted in higher collagen deposition, which could be related to a slower activity during the remodelling phase [57, 58]. Overall, the cell-loaded decellularised matrices showed higher regenerative capacity over the cell-loaded collagen/GAG scaffold, which is in agreement with previous publications with that have shown decellularised porcine small intestinal submucosa and dermis to promote ADSC production of immunomodulatory (e.g. TGF-β, COX-2) [54] and angiogenic (VEGF, FGF-2) molecules and to reduce inflammation (e.g. IL-6, iNOS) markers [23, 54]. It is worth noting that angiogenesis was particularly enhanced when hADSCs were delivered through the BM side of XenoMEM™. Again, we believe that compositional differences may be responsible for this, considering that previous studies have shown improved angiogenetic capacity of scaffolds loaded with basement membrane components [59, 60].

## Conclusion

In the quest of the ideal biomaterial for adipose-derived stem cell delivery in a wound healing scenario, this study demonstrated the capacity of extracellular matrix-biomaterials to achieve higher cell localisation at the side of implantation than direct injections, even though they (the biomaterials) were loaded with half of the cells. Further, the combined biofunctionality of the extracellular matrix-biomaterials and the stem cells resulted in enhanced regenerative capacity. Collectively, our data further support the use of extracellular matrix-based biomaterials, in particular decellularised porcine peritoneum, as adipose-derived stem cell carriers in a wound healing scenario.

## Supplementary Information

**Additional file 1: Supplementary Figure S1**.Cytoskeleton (red) and nuclei (blue) staining of human ADSCs showed the lower proliferation of cells on Integra™ Matrix Wound Dressing, whilst on the tissue grafts it appeared to be higher, particularly on their BM sides. Scale bars 100 μm. **Supplementary Figure S2.** Calcein (green) and ethidium homodimer (red) staining of alive and dead cells, respectively, revealed human ADSCs viability to be unaffected in any of the conditions and time points. Scale bars 100 μm. **Supplementary Figure S3.**Flow cytometry analysis revealed that most (> 99%) of the human ADSCs were positive for the CD90, CD44 and CD73 markers and negative for the CD45 marker independently of the condition and at both timepoints. **Supplementary Figure S4**.Alizarin red staining of human ADSCs on TCP (**A**) after osteogenic differentiation showed deposition of calcium after 14 and 21 days, confirming the suitability of the differentiation protocol. Quantification of deposited calcium (**B**) showed a significantly increase of calcium deposition after 21 days in all conditions, although it was not significant on the Integra™ Matrix Wound Dressing. Scale bars 100 μm. ** indicates a significantly (*p* < 0.05) higher value than the TCP group. **Supplementary Figure S5.** Oil red staining of human ADSCs on TCP (**A**) after adipogenic differentiation showed the accumulation of lipids after 7, 14 and 21 days, confirming the suitability of the differentiation protocol. Analysis of released lipids by OD (**B**) revealed a significant increase of lipids deposition in all conditions after 14 days, although this was not significant on the Integra™ Matrix Wound Dressing. Scale bars 100 μm. Data presented as average ± standard deviation (*n* = 3). * indicates a significantly (*p* < 0.05) lower value than the TCP group, ** indicates a significantly (*p* < 0.05) higher value than the TCP group. **Supplementary Figure S6.** Alcian blue and fast red staining of pellets (**A**) after chondrogenic differentiation showed shrinking of the pellet and a denser deposition of GAG (blue), confirming the suitability of the differentiation protocol. GAG quantification of hADSCs under differentiation (**B**) showed a significant increase in GAG deposition on the BM sides of MatriStem™ and XenoMEM™ and a collapsed pellet hADSCs-sheet structure was observed (**C**). Scale bars 100 μm. Data presented as average ± standard deviation (*n* = 3). ** indicates a significantly (*p* < 0.05) higher value than the TCP group. **Supplementary Figure S7.** Histology analysis showed occasionally some remnants of materials that were not completely absorbed. Scale bars 200 μm.

## Data Availability

The datasets used and/or analysed during the current study are available from the corresponding author on reasonable request.

## Abbreviations

α-MEM: Alpha-minimal essential medium
BM: Basement membrane
CT: Connective tissue
DMEM: Dulbecco’s modified Eagle medium
EDTA: Ethylenediaminetetraacetic acid
ECM: Extracellular matrix
FGF-2: Fibroblast growth factor 2
FBS: Foetal bovine serum
GAG: Glycosaminoglycan
H&E: Haematoxylin/eosin
hADSCs: Human adipose-derived stem cells
ITS+1: Insulin, transferrin, sodium selenite, linoleic-bovine serum albumin
Masson’s TC: Masson’s trichrome
OD: Optical density
PFA: Paraformaldehyde
PS: Penicillin/streptomycin
PBS: Phosphate-buffered saline
TCP: Tissue culture plastic
TGF-β3: Transforming growth factor β3
VEGF: Vascular endothelial growth factor
