# Supplementary material for: Extracellular matrix-based biomaterials as adipose-derived stem cell delivery vehicles in wound healing: a comparative study between a collagen scaffold and two xenografts
Source: Stem Cell Res Ther. 2020 Nov 27;11:510. doi: 10.1186/s13287-020-02021-x (PMC7694925; doi:10.1186/s13287-020-02021-x)

# Supplementary Information

# Title

Extracellular matrix-based biomaterials as adipose derived stem cell delivery vehicles in wound healing: A comparative study between a collagen scaffold and two xenografts

# Authors

Héctor Capella-Monsonís (1, 2), Andrea De Pieri (1, 2, 3), Rita Peixoto (1, 2), Stefanie Korntner (1, 2), Dimitrios I. Zeugolis* (1, 2, 4)

# Affiliations

(1) Regenerative, Modular & Developmental Engineering Laboratory (REMODEL), Biomedical Sciences Building, National University of Ireland Galway (NUI Galway), Galway, Ireland

(2) Science Foundation Ireland (SFI) Centre for Research in Medical Devices (CÚRAM), Biomedical Sciences Building, National University of Ireland Galway (NUI Galway), Galway, Ireland

(3) Proxy Biomedical Ltd, Spiddal, Ireland

(4) Regenerative, Modular & Developmental Engineering Laboratory (REMODEL), Faculty of Biomedical Sciences, Università della Svizzera Italiana (USI), Lugano, Switzerland

* Corresponding Author: Dimitrios I. Zeugolis, REMODEL, NUI Galway & USI. Telephone: +41 58 666 40 00; Email: dimitrios.zeugolis@usi.ch

**Supplementary Figure S1:** Cytoskeleton (red) and nuclei (blue) staining of human ADSCs showed the lower proliferation of cells on Integra™ Matrix Wound Dressing, whilst on the tissue grafts it appeared to be higher, particularly on their BM sides. Scale bars 100 *μ*m.


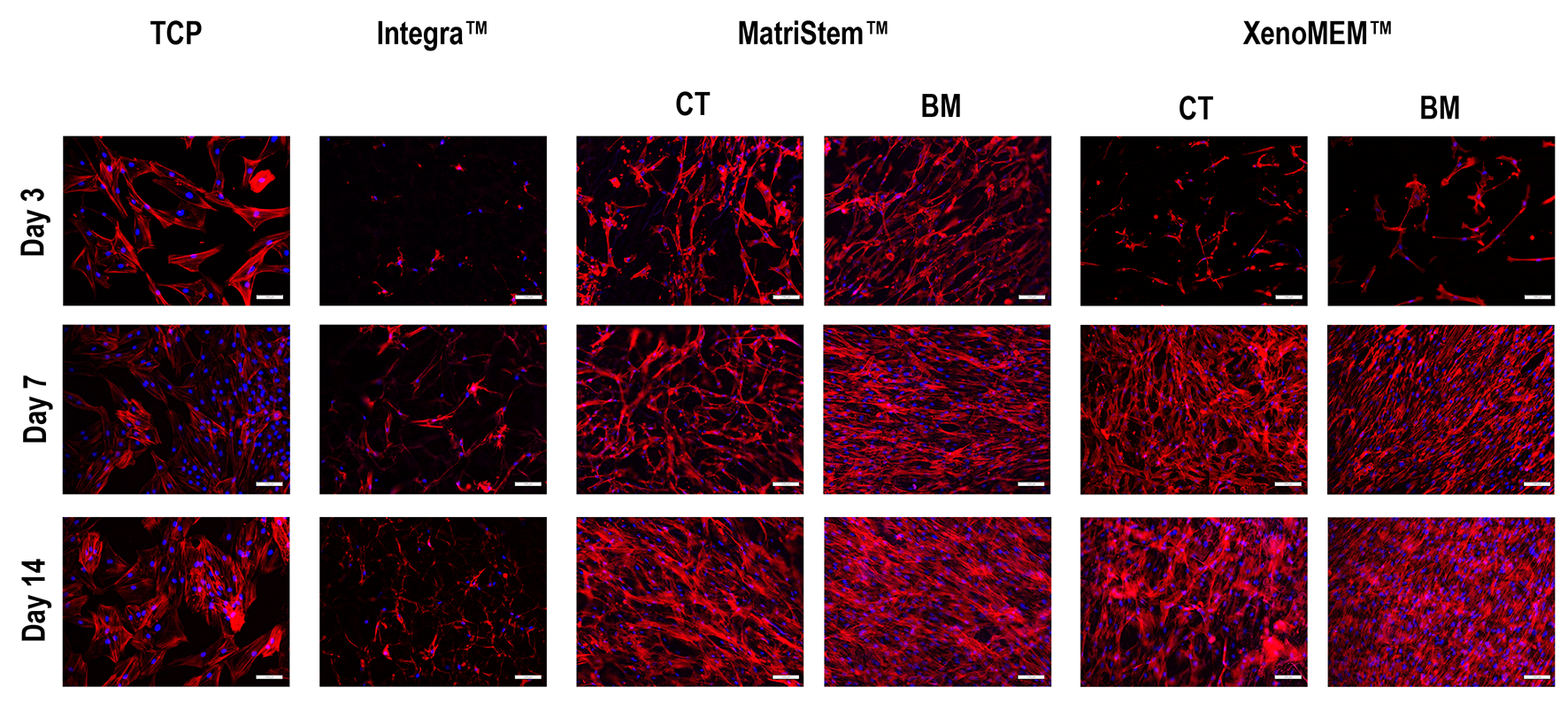


**Supplementary Figure S2:** Calcein (green) and ethidium homodimer (red) staining of alive and dead cells, respectively, revealed human ADSCs viability to be unaffected in any of the conditions and time points. Scale bars 100 *μ*m.


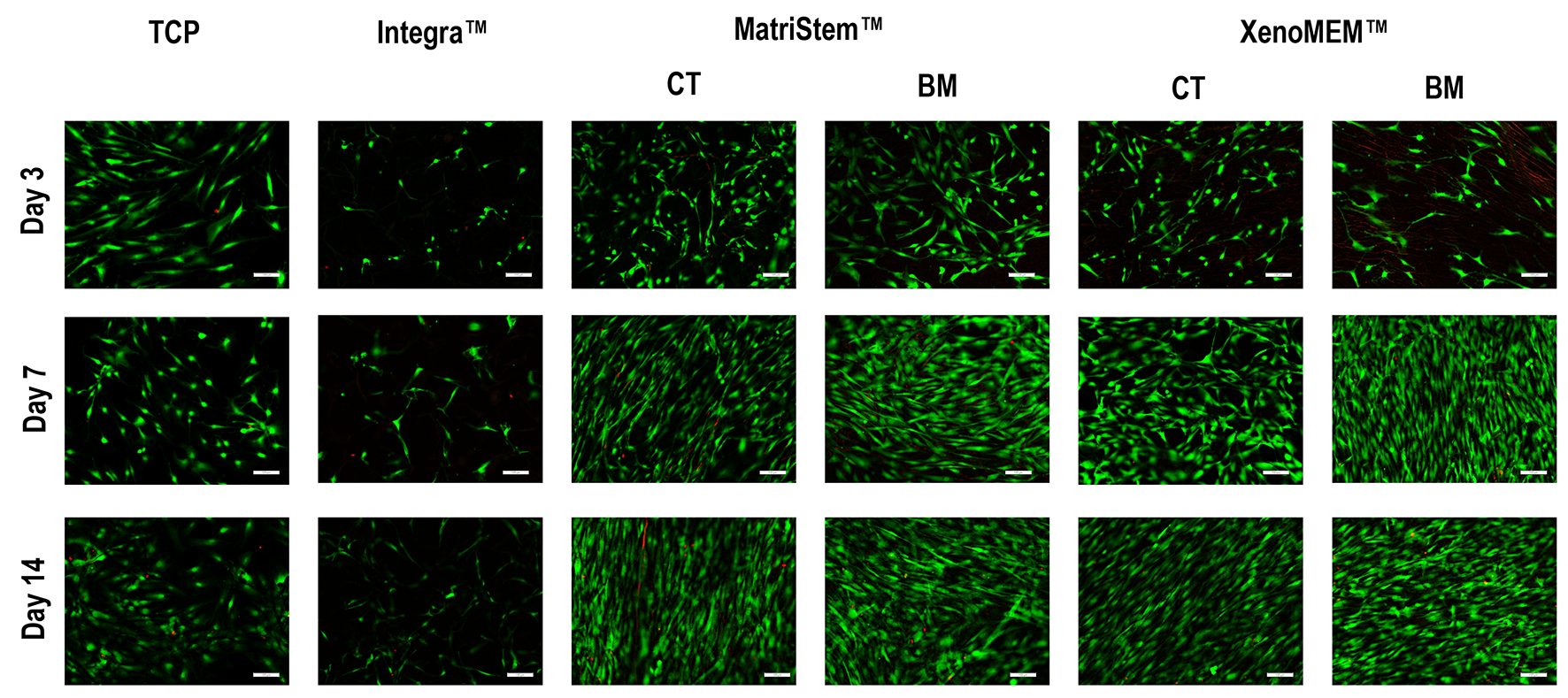


**Supplementary Figure S3:** Flow cytometry analysis revealed that most (> 99 %) of the human ADSCs were positive for the CD90, CD44 and CD73 markers and negative for the CD45 marker independently of the condition and at both timepoints.


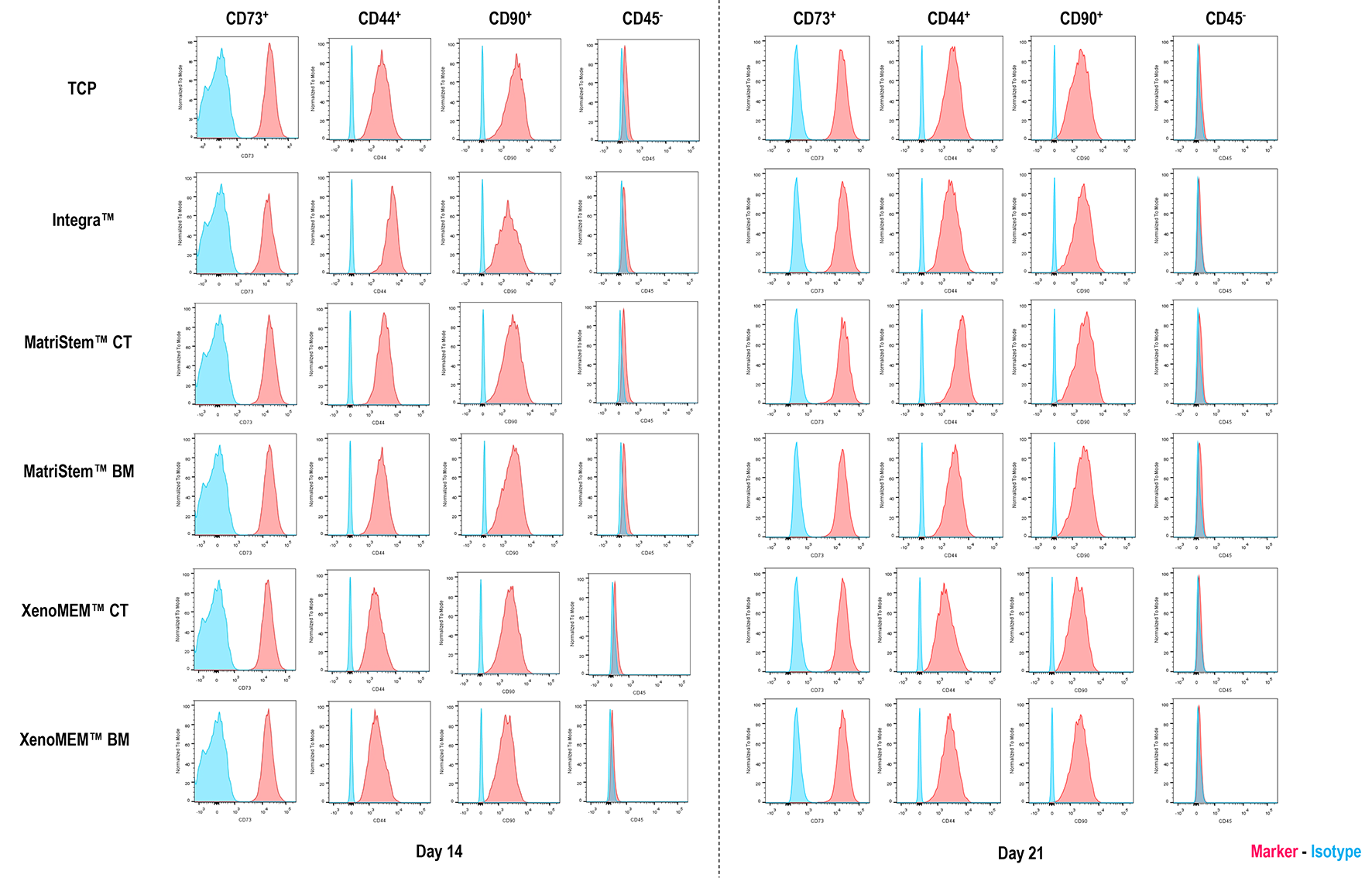


**Supplementary Figure S4:** Alizarin red staining of human ADSCs on TCP (**A**) after osteogenic differentiation showed deposition of calcium after 14 and 21 days, confirming the suitability of the differentiation protocol. Quantification of deposited calcium (**B**) showed a significantly increase of calcium deposition after 21 days in all conditions, although it was not significant on the Integra™ Matrix Wound Dressing. Scale bars 100 *μ*m. ** indicates a significantly (*p* < 0.05) higher value than the TCP group.


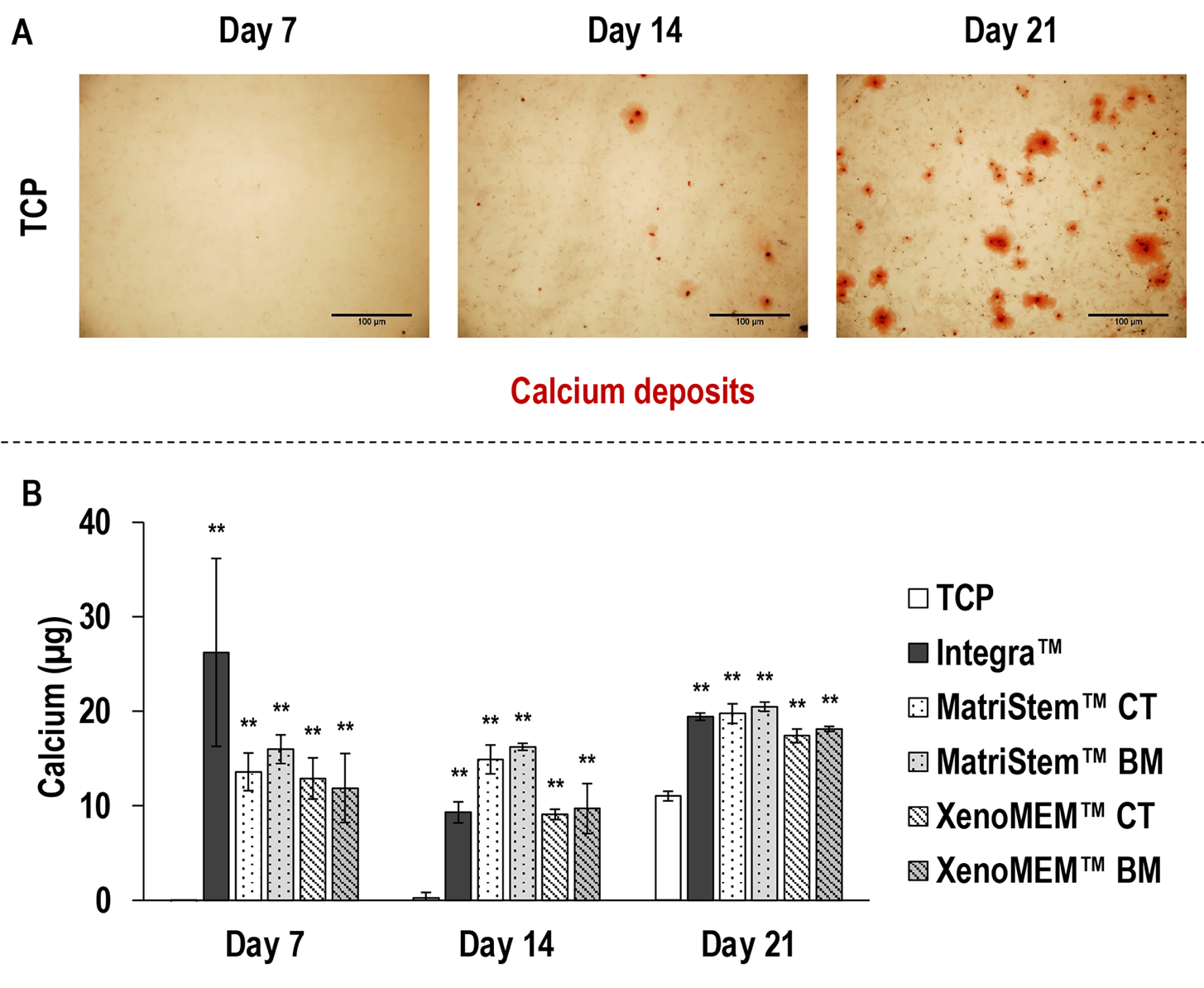


**Supplementary Figure S5:** Oil red staining of human ADSCs on TCP (**A**) after adipogenic differentiation showed the accumulation of lipids after 7, 14 and 21 days, confirming the suitability of the differentiation protocol. Analysis of released lipids by OD (**B**) revealed a significant increase of lipids deposition in all conditions after 14 days, although this was not significant on the Integra™ Matrix Wound Dressing. Scale bars 100 *μ*m. Data presented as average ± standard deviation (n=3). * indicates a significantly (*p* < 0.05) lower value than the TCP group, ** indicates a significantly (*p* < 0.05) higher value than the TCP group.


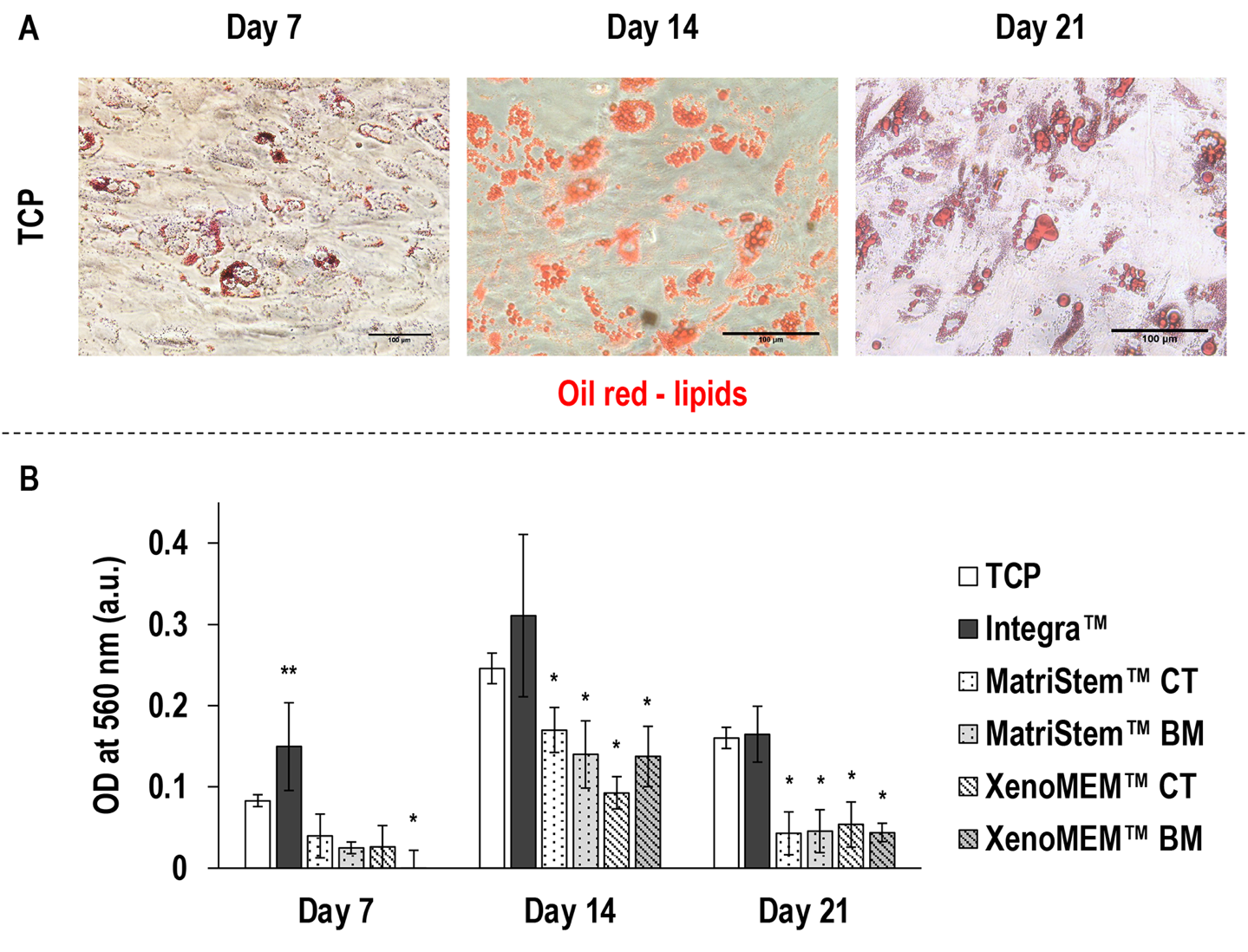


**Supplementary Figure S6:** Alcian blue and fast red staining of pellets (**A**) after chondrogenic differentiation showed shrinking of the pellet and a denser deposition of GAG (blue), confirming the suitability of the differentiation protocol. GAG quantification of hADSCs under differentiation (**B**) showed a significant increase in GAG deposition on the BM sides of MatriStem™ and XenoMEM™ and a collapsed pellet hADSCs-sheet structure was observed (**C**). Scale bars 100 *μ*m. Data presented as average ± standard deviation (n=3). ** indicates a significantly (*p* < 0.05) higher value than the TCP group.


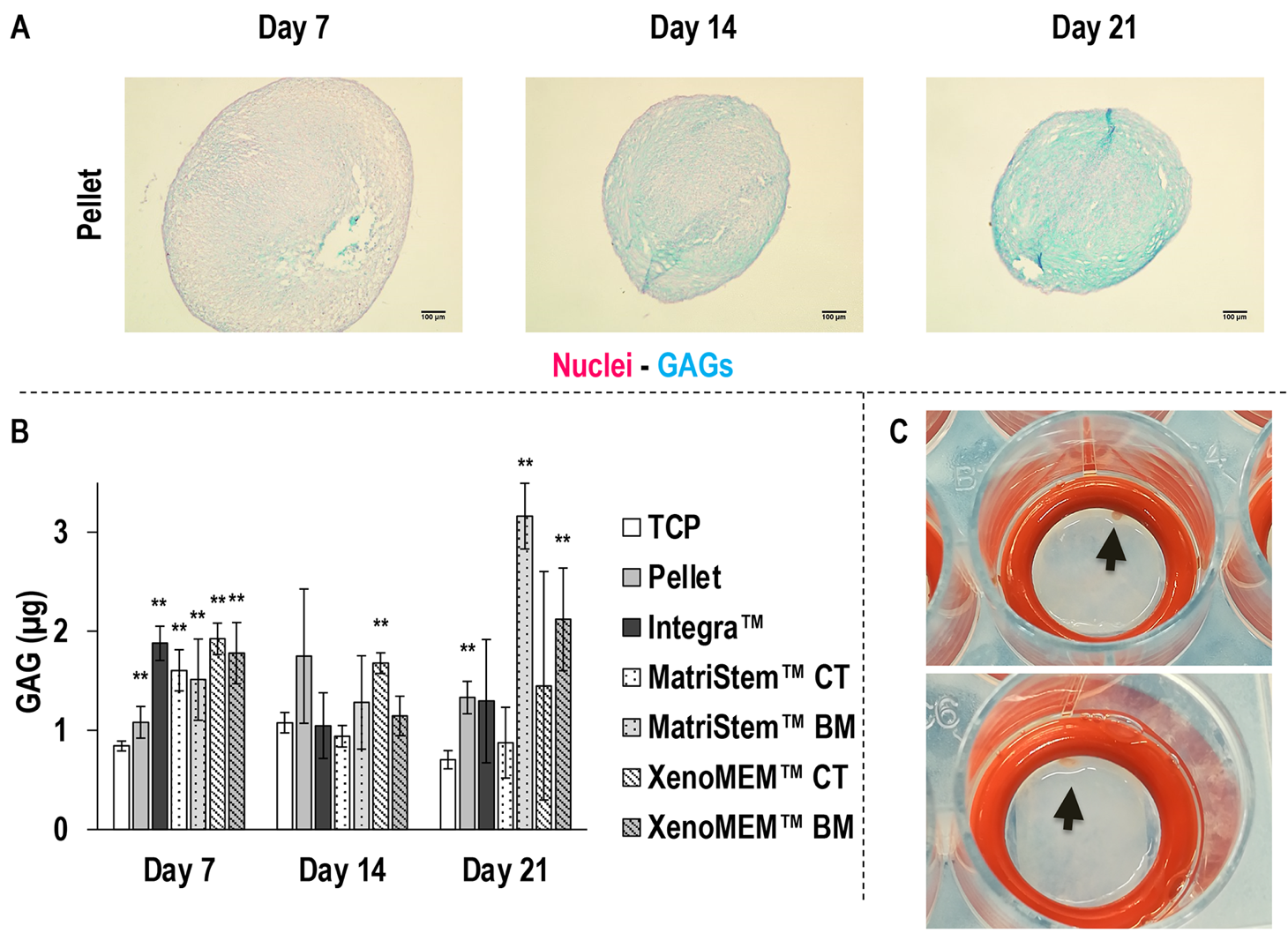


**Supplementary Figure S7:** Histology analysis showed occasionally some remnants of materials that were not completely absorbed. Scale bars 200 *μ*m.


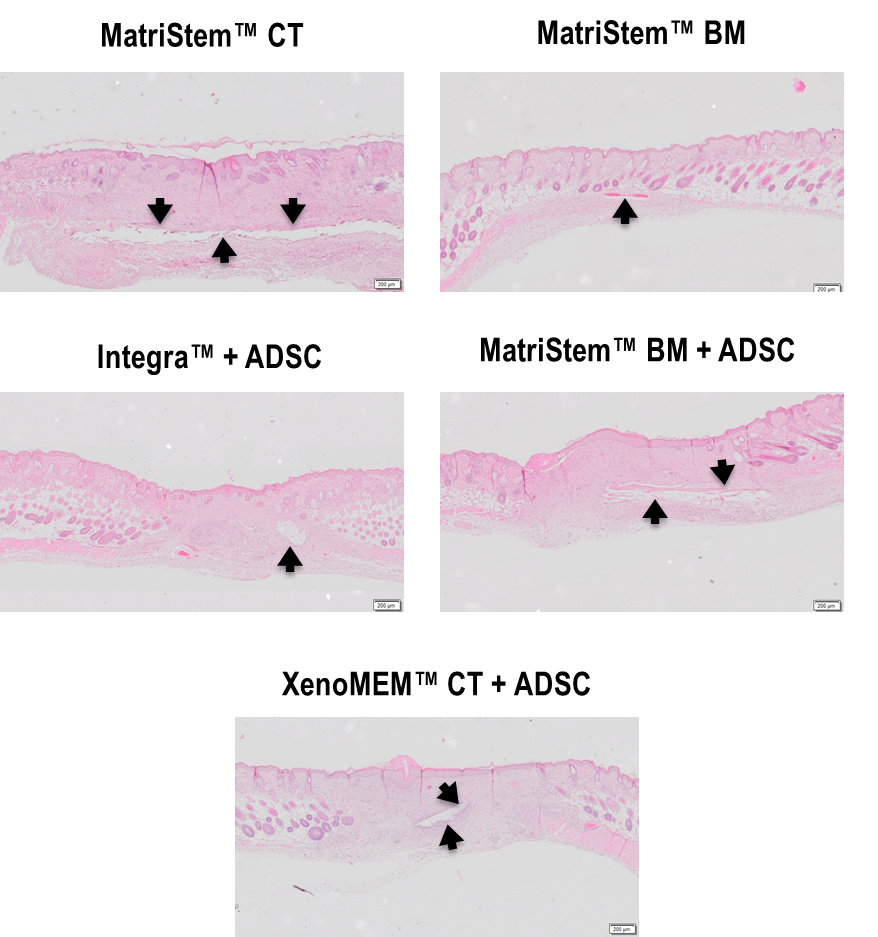

Supplement: Supplementary file 1 — Additional file 1: Supplementary Figure S1.Cytoskeleton (red) and nuclei (blue) staining of human ADSCs showed the lower proliferation of cells on Integra™ Matrix Wound Dressing, whilst on the tissue grafts it appeared to be higher, particularly on their BM sides. Scale bars 100 μm. Supplementary Figure S2. Calcein (green) and ethidium homodimer (red) staining of alive and dead cells, respectively, revealed human ADSCs viability to be unaffected in any of the conditions and time points. Scale bars 100 μm. Supplementary Figure S3.Flow cytometry analysis revealed that most (> 99%) of the human ADSCs were positive for the CD90, CD44 and CD73 markers and negative for the CD45 marker independently of the condition and at both timepoints. Supplementary Figure S4.Alizarin red staining of human ADSCs on TCP (A) after osteogenic differentiation showed deposition of calcium after 14 and 21 days, confirming the suitability of the differentiation protocol. Quantification of deposited calcium (B) showed a significantly increase of calcium deposition after 21 days in all conditions, although it was not significant on the Integra™ Matrix Wound Dressing. Scale bars 100 μm. ** indicates a significantly (p < 0.05) higher value than the TCP group. Supplementary Figure S5. Oil red staining of human ADSCs on TCP (A) after adipogenic differentiation showed the accumulation of lipids after 7, 14 and 21 days, confirming the suitability of the differentiation protocol. Analysis of released lipids by OD (B) revealed a significant increase of lipids deposition in all conditions after 14 days, although this was not significant on the Integra™ Matrix Wound Dressing. Scale bars 100 μm. Data presented as average ± standard deviation (n = 3). * indicates a significantly (p < 0.05) lower value than the TCP group, ** indicates a significantly (p < 0.05) higher value than the TCP group. Supplementary Figure S6. Alcian blue and fast red staining of pellets (A) after chondrogenic differentiation showed [file 13287_2020_2021_MOESM1_ESM.docx]
